# Percutaneous cryoablation: a novel treatment option in non-visceral metastases of the abdominal cavity after prior surgery

**DOI:** 10.1007/s00261-022-03598-y

**Published:** 2022-07-02

**Authors:** D. J. van der Reijd, T. R. Baetens, F. Gomez Munoz, B. M. Aarts, M. J. Lahaye, N. M. Graafland, C. A. R. Lok, A. G. J. Aalbers, N. F. M. Kok, R. G. H. Beets-Tan, M. Maas, E. G. Klompenhouwer

**Affiliations:** 1 Department of Radiology, The Netherlands Cancer Institute, Amsterdam, The Netherlands; 2 GROW School for Oncology and Developmental Biology, Maastricht University Medical Centre, Maastricht, The Netherlands; 3 Department of Urology, The Netherlands Cancer Institute, Amsterdam, The Netherlands; 4 Department of Gynecology, The Netherlands Cancer Institute, Amsterdam, The Netherlands; 5 Department of Surgery, The Netherlands Cancer Institute, Amsterdam, The Netherlands; 6 Faculty of Health Sciences, University of Southern Denmark, Odense, Denmark; 7 Department of Interventional Radiology, Hospital Clinic Universitari, Barcelona, Spain

**Keywords:** Cryosurgery, Abdomen, Neoplasms, Metastases, Interventional Radiology

## Abstract

**Purpose:**

To assess the primary safety and oncological outcome of percutaneous cryoablation in patients with non-visceral metastases of the abdominal cavity after prior surgery.

**Methods:**

All patients with non-visceral metastases after prior abdominal surgery, treated with percutaneous cryoablation, and at least one year of follow-up were retrospectively identified. Technical success was achieved if the ice-ball had a minimum margin of 10 mm in three dimensions on the per-procedural CT images. Complications were recorded using the Society of Interventional Radiology (SIR) classification system. Time until disease progression was monitored with follow-up CT and/or MRI. Local control was defined as absence of recurrence at the site of ablation.

**Results:**

Eleven patients underwent cryoablation for 14 non-visceral metastases (mean diameter 20 ± 9 mm). Primary tumor origin was renal cell (*n* = 4), colorectal (*n* = 3), granulosa cell (*n* = 2), endometrium (*n* = 1) and appendix (*n* = 1) carcinoma. Treated metastases were localized retroperitoneal (*n* = 8), intraperitoneal (*n* = 2), or in the abdominal wall (*n* = 4). Technical success was achieved in all procedures. After a median follow-up of 27 months (12–38 months), all patients were alive. Local control was observed in 10/14 non-visceral metastases, and the earliest local progression was detected after ten months. No major adverse events occurred. One patient suffered a minor asymptomatic adverse event.

**Conclusion:**

This proof-of-concept study suggests that cryoablation can be a minimal invasive treatment option in a selected group of patients with non-visceral metastases in the abdominal cavity after prior surgery.

**Graphical abstract:**

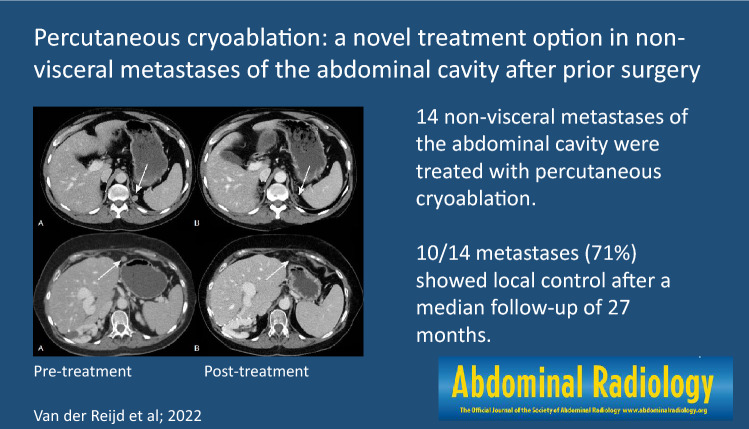

## Introduction

Abdominal cancers are plagued by a high incidence of recurrences, especially in advanced-stage disease. Tumor recurrence may be due to local recurrence or arise elsewhere in the abdominal cavity. For instance, in patients treated with curative intent for colorectal cancer (CRC), overall recurrence occurs in 17% to 25% [1–3]. The recurrence rate in renal cell cancer (RCC) is 21–27% [4–6]. Recurrence rates in granulosa cell and endometrial cancers are strongly dependent on stage. Curative treatment of recurrences can be challenging; it aims (1) to improve overall survival, (2) reduce tumor burden, or (3) avoid or delay the start of systemic therapy. Surgery with curative intent is usually performed provided the recurrent disease is limited. However, not all patients are eligible for surgery due to location, proximity to critical structures that can potentially be damaged, extensive prior surgery, morbidity or extent of disease. Besides, patients with an extensive oncological history might benefit from the advantages of minimally invasive therapy, such as the reduced risk of morbidity, shorter hospital admissions, and reduced pain and recovery time.

While heat or cold-based thermal ablation techniques are well-known local treatment options for lesions in the abdominal viscera (e.g., liver/kidney) [7–10], the clinical benefit of thermal ablation in non-visceral metastases of the abdominal cavity has not yet been established. Cryoablation is a thermal ablation technique wherein tumor tissue is destroyed by extremely cold temperatures. With cryoablation, probes inserted in the tumor cause freezing, resulting in mechanical damage, dehydration and local ischemia, leading to cell death [9, 11]. The formation of an ice-ball creating the ablation zone can be visualized during the freezing process. This real-time visualization limits the risk of damage to adjacent structures and is an important advantage of cryoablation over other thermal ablation techniques.

The aim of this proof-of-concept study is to assess the safety and oncological outcome of percutaneous cryoablation in patients with limited non-visceral metastases of the abdominal cavity after prior surgery.

## Materials and methods

### Patients

Our Institutional Review Board approved this single-center, retrospective study, and informed consent was waived. From November 2018 to January 2021, all patients treated with cryoablation for non-visceral metastases of the abdominal cavity were included after a follow-up of at least one year. The decision to perform cryoablation was made by a multidisciplinary tumor board (MDT) consisting of a medical oncologist, radiologist, surgical oncologist (urologist, gastrointestinal surgeon, or gynecologist), radiation oncologist, and interventional radiologist. Whole-body CT imaging was performed in all patients to rule out widespread disease. Patients considered eligible for cryoablation by the MDT, had confined non-visceral metastases in the abdominal cavity after prior abdominal surgery, based on pathological proof and/or growth on consecutive imaging. Patients with widespread disease or voluminous non-visceral metastases were not considered eligible. Patient demographics and tumor characteristics, including primary origin, location, and prior local and systemic treatment were recorded.

### Cryoablation

All cryoablation procedures were performed, or supervised, by an expert interventional radiologist with 4 to 12 years of experience in thermal ablation. General or epidural anesthesia was administered, and computed tomography (CT) guidance (CT Somatom Sensation Open, Siemens®, Munchen, Germany) was used for realtime evaluation of the procedure. Cryoablation was performed using the Visual ICE™ system (Boston Scientific, USA) with IceForce® or IcePearl® cryoprobes. The number of cryoprobes was determined by the size of the lesion, and inserted with 1 to 2 cm spacing. If deemed necessary, air- or hydrodissection was applied to protect adjacent structures using room air or 5% glucose solution combined with iodine contrast. At least two cycles of freezing, passive thawing, and active thawing were completed with a minimum time of 10 min, 2 min, and 2 min, respectively. Technical success was achieved if the ice-ball had a minimum margin of 10 mm in three dimensions on the periprocedural CT images. Patients stayed overnight for monitoring and were discharged the day after the cryoablation procedure.

### Follow-up

Follow-up imaging was scheduled at least at one, six, and twelve months after treatment or when clinical symptoms occurred. The time until disease progression, both local and distant, was monitored using CT and/or magnetic resonance imaging (MRI). Local control was defined as the absence of tumor regrowth within 1 cm of the ablation zone. Distant progression included both new tumor foci at distant sites from the ablation zone and progression of initially stable metastases. If disease progression occurred, further treatment options were discussed in the MDT, and data about the time between ablation and the start of systemic treatment were collected. Adverse events were recorded using the Society of Interventional Radiology (SIR) classification system.

### Analysis

Descriptive statistics were used to report on the results. Categorical variables were expressed as frequencies and percentages. Continuous variables were reported as mean and standard deviation or median and range, depending on the distribution of the data.

## Results

A total of 11 patients underwent cryoablation for 14 non-visceral metastases. Metastases originated from renal cell (*n* = 4), colorectal (*n* = 3), granulosa cell (*n* = 2), endometrium (*n* = 1), and appendix (*n* = 1) carcinoma; patient details are displayed in Table 1. Treated metastases were localized retroperitoneal (*n* = 8), intraperitoneal (*n* = 2), or in the abdominal wall (*n* = 4). Patients were treated for one up to three lesions. The non-visceral metastases had a mean longest diameter of 20 ± 9 mm. 10/11 patients had histopathological evidence available for metastasized disease. Of the treated non-visceral metastases, 6/14 were confirmed by biopsy. Technical success was achieved in all lesions. No major adverse events occurred. One patient developed an asymptomatic pseudocyst adjacent to the ablation zone without the need for further treatment (SIR grade A). Treatment and outcome details are presented in Table 2.

**Table 1:** Patient demographics and characteristics

Patient	Age	Sex	Primary	Comorbidities	Metastases location	Other metastases^a^	Prior surgery	Prior CTx
1	57	F	Appendix		Diaphragm		Right hemicolectomy, debulking with HIPEC (2x), liver resection	Yes
2	67	F	CRC		Perirenal space		Right hemicolectomy with enbloc gastric resection, debulking with HIPEC (2x), abdominal wall metastasectomy	Yes
3	77	M	CRC	TIA	Gerota’s fascia (3x)	Adrenal	Left hemicolectomy, resection local recurrence, debulking with HIPEC	No
4	78	M	CRC	DM	Abdominal wall, Gerota’s fascia		Left hemicolectomy, resection local recurrence, lymph node dissection	Yes
5	57	M	RCC		Pancreas tail		Nephrectomy	No
6	56	M	RCC		Perirenal space	Lung	Nephrectomy, lymph node dissection	No
7	76	F	RCC		Abdominal wall		Nephrectomy	No
8	61	M	RCC	CABG, CRC	Gerota’s fascia	Lung	Partial nephrectomy, abdominoperineal resection	No
9	75	F	Endometrium		Abdominal wall		Hysterosalpingo-oophorectomy, debulking	Yes
10	67	F	GCT		Epigastrium		Salpingo-oophorectomy and sigmoid resection, right hemicolectomy, debulking (4x), liver resection	Yes
11	75	F	GCT		Abdominal wall		Oophorectomy, debulking, lymph node dissection (2x), abdominal wall metastasectomy	No

*CABG* coronary artery bypass grafting, *CRC* colorectal cancer, *CTx* chemotherapy treatment, *DM* diabetes mellitus, *F* female, *GCT* granulosa cell tumor, *HIPEC* hyperthermic intraperitoneal chemotherapy, *M* male, *RCC* renal cell cancer, *TIA* transient ischaemic attack

^a^At time of cryoablation

**Table 2:** Treatment details and outcome

Pt	Metastases location	Peritoneal location	Size (cm)	Number of probes	Dissection	Anesthesia	Adverse events	Follow-up (m)	Alive/deceased	Time to local progression (m)	Time to systemic progression (m)	Time to systemic treatment (m)
1	Diaphragm	Intraperitoneal	3,2	3	-	General	-	36	Alive	-	-	-
2	Perirenal space	Retroperitoneal	2,0	2	Air	Epidural	-	30	Alive	-	9	16
3^a^	Gerota’s fascia	Retroperitoneal	1,6	1	-	Epidural	-	32	Alive	10	19	-
	Gerota’s fascia	Retroperitoneal	3,7	2	Hydro	General	-	15	Alive	10	4	-
	Gerota’s fascia	Retroperitoneal	1,1	1	-	General	-	15	Alive	-	4	-
4^a^	Abdominal wall	Abdominal wall	1,3	1	Air	Epidural	-	17	Alive	-	-	-
	Gerota’s fascia	Retroperitoneal	2,1	2	-	Epidural	-	17	Alive	-	-	-
5	Pancreatic tail	Retroperitoneal	3,9	2	Hydro	General	Minor^b^	32	Alive	-	-	-
6	Perirenal space	Retroperitoneal	1,6	1	Air	Epidural	-	38	Alive	-	1	2
7	Abdominal wall	Abdominal wall	2,2	1	Hydro	Epidural	-	24	Alive	14	-	-
8	Gerota’s fascia	Retroperitoneal	1,5	1	-	General	-	13	Alive	-	5	14
9	Abdominal wall	Abdominal wall	1,1	1	Air	Epidural	^−^	30	Alive	13	-	-
10	Epigastrium	Intraperitoneal	1,3	1	Hydro	Epidural	-	30	Alive	-	27	-
11	Subcutaneous	Abdominal wall	1,2	1	Hydro	Epidural	-	12	Alive	-	-	-

^a^Multiple lesions treated per patient

^b^Pseudocyst

### Follow-up

**Fig. 1:**
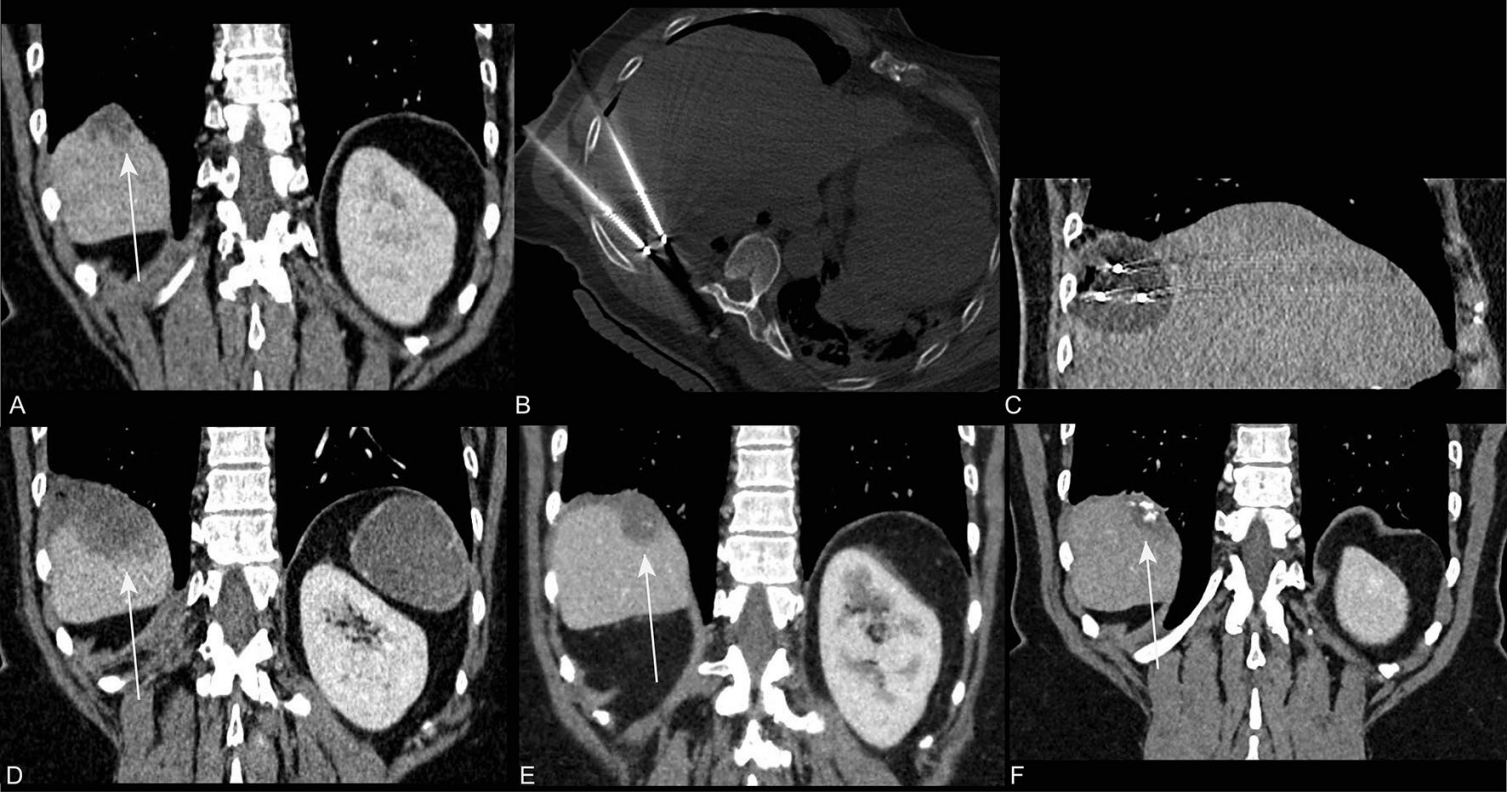
A 57-year-old female patient with a history of mucinous appendix carcinoma treated with a right hemicolectomy, two debulkings including HIPEC, and a liver metastectomy, now presents with a rise in carcinoembryonic antigen (CEA) from 8.0 to 38 µg/L. CT shows a peritoneal metastases of 32 mm located on the diaphragm invading the liver (**A**). Cryoablation was performed using three needles (**B**; transverse CT, **C**; sagittal CT). Coronal CT images show complete ablation after one month (**D**), and local control after 5 months (**E**) and 3 years (**F**) with a stable CEA varying between 5.6 and 7.0 µg/L

**Fig. 2:**
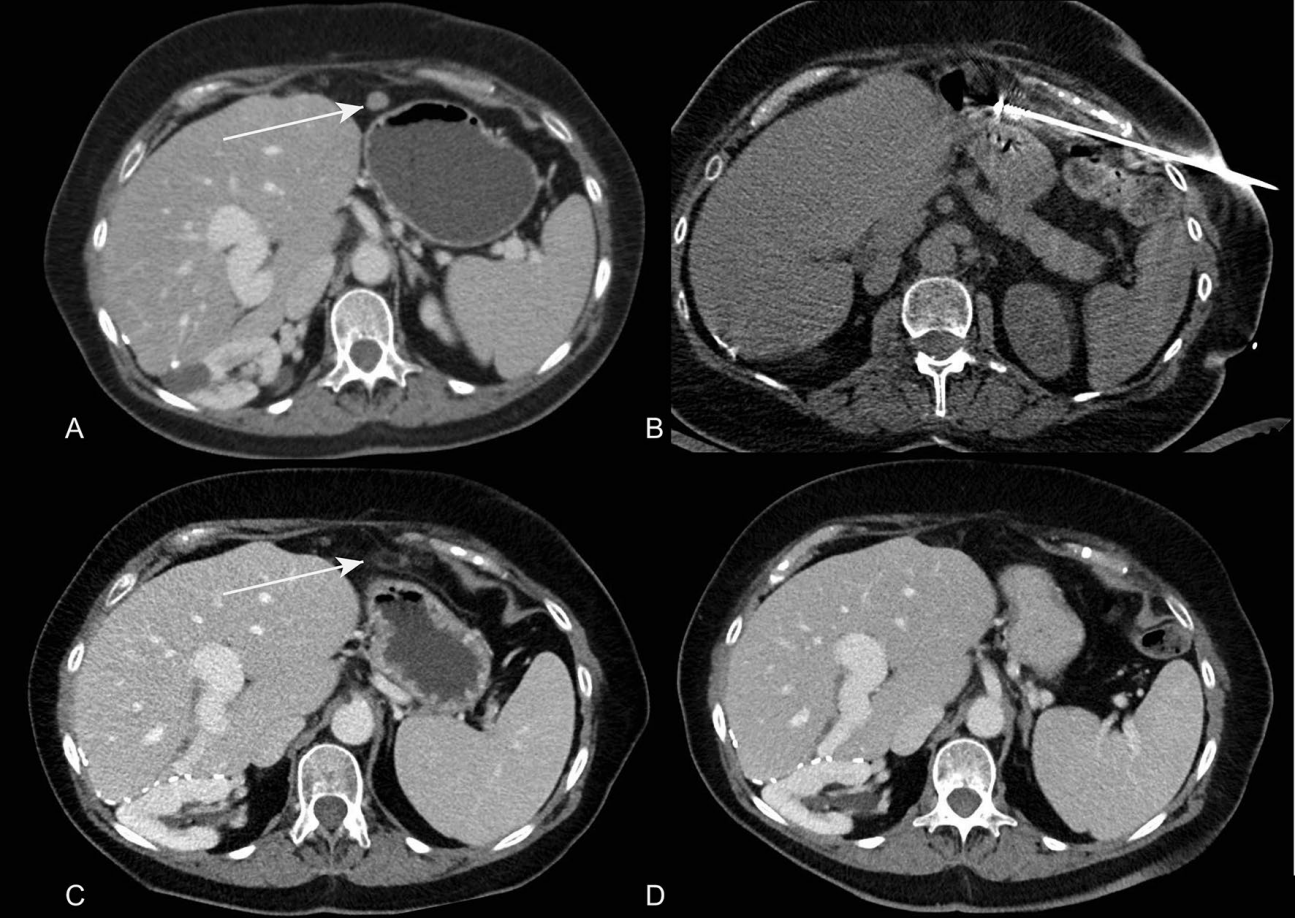
A 67-year-old female patient presents with a peritoneal metastases of granulosa cell carcinoma in the epigastric region of 13 mm (**A**). Cryoablation with one needle with hydrodissection for stomach proximity (**B**). Follow-up CT shows complete ablation after one month (**C**) and persistent local control after 27 months (**D**)

**Fig. 3:**
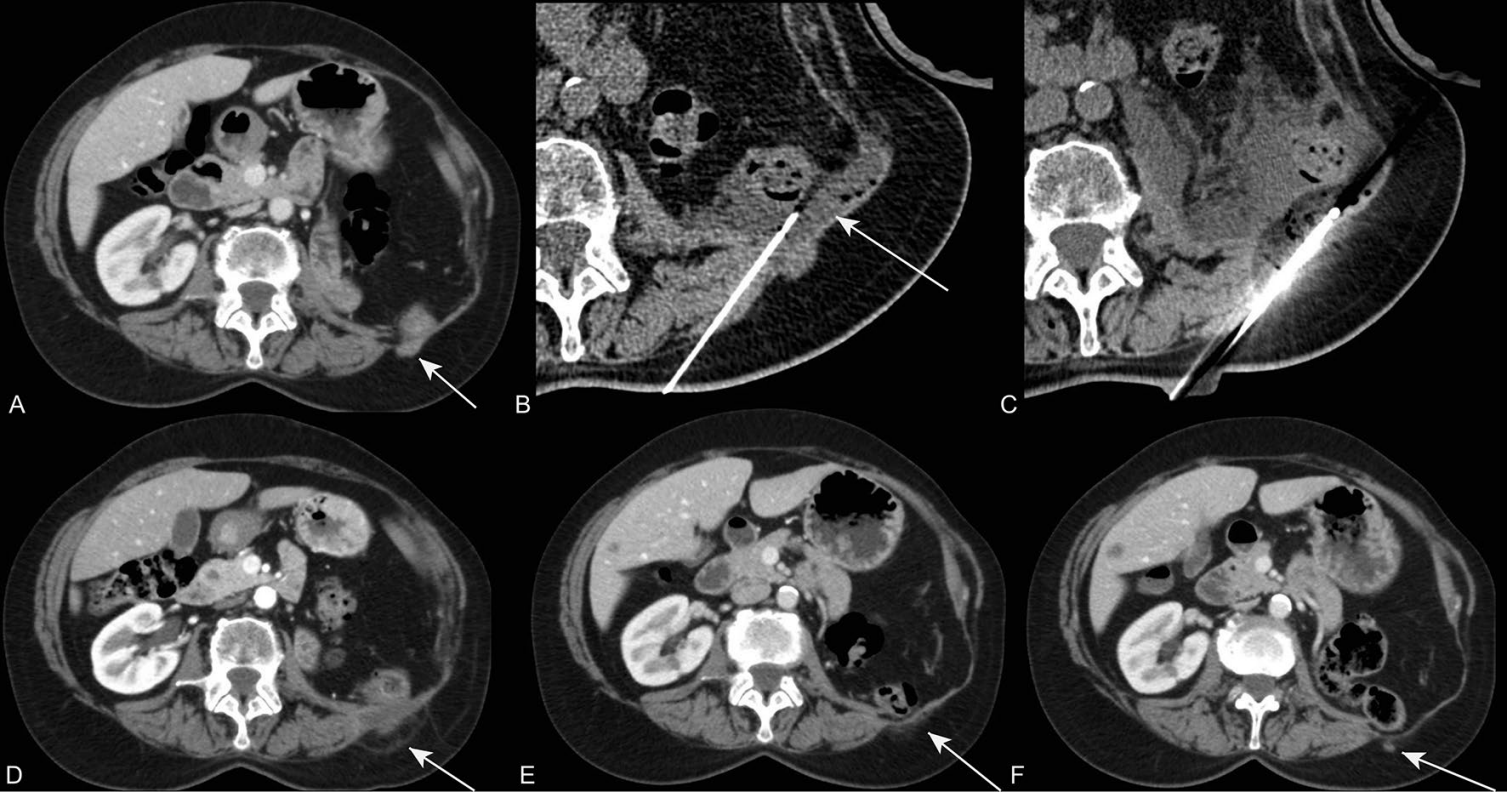
A 76-year-old female patient with a non-visceral metastases of RCC in the abdominal wall of 22 mm (**A**) after nephrectomy, which was previously treated with SBRT and twice with radiofrequency ablation. Hydrodissection (**B**) was performed for bowel proximity and one needle was used during cryoablation (**C**). Follow-up CT shows complete ablation after one month (**D**), and persistent local control after 8 months (**E**). Local tumor progression was detected after 14 months (**F**) which was left untreated and closely monitored with watchful waiting

All patients were alive after a median follow-up of 27 months (range 12–38 months), and 10/14 metastases (71%) showed persistent local control. Figures 1 and 2 show examples of successfully treated lesions. The four metastases with local progression were found in three patients (patients 3, 7 and 9 (Table 2)). One patient had local progression and was re-treated with cryoablation 16 months after the initial procedure. Local progression reoccurred ten months after the second cryoablation procedure. The two other patients with local progression had single abdominal wall metastases at the time of the cryoablation. Prior treatment included resection and radiotherapy in one patient, and radiotherapy and radiofrequency ablation in the other patient (patients 9 and 7, respectively). Local control was obtained for 13 and 14 months after cryoablation. Figure 3 displays the images of a patient with local progression.

Five patients developed distant progression outside the ablation zone during follow-up, including one patient who also had local progression. Three out of these five patients had other distant metastases at the time of the cryoablation procedure and the cryoablation was performed for oligoprogression. Three patients started with systemic treatment after distant progression, and the time between the cryoablation procedure and the start of systemic treatment was 2, 14, and 16 months.

## Discussion

The present study aims to assess the safety and oncological outcome of this novel strategy in which percutaneous cryoablation was used for patients with limited non-visceral metastases of the abdominal cavity after prior abdominal surgery. This proof-of-concept study demonstrated that percutaneous cryoablation could be a treatment option in carefully selected patients. These patients were treated for a maximum of three non-visceral metastases ranging from 1.1 to 3.9 cm. We observed good local control in 71% of the patients. No major adverse events occurred. One minor adverse event did not require treatment. This suggests cryoablation is a safe procedure for non-visceral abdominal metastases.

Although adequate ablative margins were achieved during the cryoablation procedures, 4 out of 14 metastases showed local progression. An explanation could be that microscopic tumor foci in the vicinity of the metastases were already present at the time of the ablation. Some studies divided local recurrences after cryoablation in procedural and satellite etiology [12, 13]. Bang et al*.* defined a procedure-related recurrence, as a recurrence in the tumor rim due to inadequate and sublethal temperatures, whereas satellite recurrences were adjacent lesions located within 1 cm of the ablation zone [12]. It suggests that some patients might have local spread of disease around the metastases, which is not visible on imaging and therefore not included in the ablation zone.

In our study, the time to local tumor progression varied between 10 to 14 months. These findings are in contrast with the results of Littrup et al., who have reported an average time to recurrence of 4 months after cryoablation of soft-tissue tumors [13]. We hypothesize, that this could be due to their mean follow-up time of 11 months (and 9 months specifically for retroperitoneal tumors), which could result in the missing of recurrences after 11 months. Other studies found comparable results with ours. Parvinian et al. reported a median time to recurrence of 11 months after cryoablation of lymph node metastases [14]. Similarly, a median progression-free survival of 10 months was found after cryoablation of recurrent CRC in the pelvic cavity [15]. These results suggest that even when local control fails, cryoablation could postpone tumor progression for up to almost a year.

Our findings regarding safety and adverse event rates align with other studies reporting on cryoablation in tumor recurrences in the abdomen. Small series reported 0 to 3% major and 0 to 7% minor adverse event rates [14, 16–18]. Whereas Wang et al. found more adverse events with 9% major and 40% minor complications after cryoablation of pelvic CRC recurrences [15]. This difference could be explained by patient selection. Some of their patients were treated to relieve pain; these patients had larger target lesions that were fixed to other structures. In their study, all patients recovered completely, confirming the safety of cryoablation in the abdominal cavity.

This study has several limitations. First, the study had a retrospective design. Second, the patient population was heterogeneous in terms of primary tumor origins, previous treatment, and localizations of treated metastases. Furthermore, histopathological evidence of metastasized disease was missing for 1/11 patients. Being limited to a small study population, this study lacks analysis of risk factors for tumor progression. Nevertheless, we advocate that this minimal invasive treatment should be discussed during MDT meetings in specific patients with small localized metastases of the non-visceral abdomen.

In conclusion, this proof-of-concept study suggests that percutaneous cryoablation can be a minimal invasive treatment option in a selected group of patients with non-visceral metastases of the abdominal cavity after prior surgery. Cryoablation should specifically be considered in patients with metastases that are limited in size and number.
